# When David Beats Goliath: The Advantage of Large Size in Interspecific Aggressive Contests Declines over Evolutionary Time

**DOI:** 10.1371/journal.pone.0108741

**Published:** 2014-09-24

**Authors:** Paul R. Martin, Cameron K. Ghalambor

**Affiliations:** 1 Department of Biology, Queen's University, Kingston, Ontario, Canada; 2 Department of Biology and Graduate Degree Program in Ecology, Colorado State University, Fort Collins, Colorado, United States of America; University of Sheffield, United Kingdom

## Abstract

Body size has long been recognized to play a key role in shaping species interactions. For example, while small species thrive in a diversity of environments, they typically lose aggressive contests for resources with larger species. However, numerous examples exist of smaller species dominating larger species during aggressive interactions, suggesting that the evolution of traits can allow species to overcome the competitive disadvantage of small size. If these traits accumulate as lineages diverge, then the advantage of large size in interspecific aggressive interactions should decline with increased evolutionary distance. We tested this hypothesis using data on the outcomes of 23,362 aggressive interactions among 246 bird species pairs involving vultures at carcasses, hummingbirds at nectar sources, and antbirds and woodcreepers at army ant swarms. We found the advantage of large size declined as species became more evolutionarily divergent, and smaller species were more likely to dominate aggressive contests when interacting with more distantly-related species. These results appear to be caused by both the evolution of traits in smaller species that enhanced their abilities in aggressive contests, and the evolution of traits in larger species that were adaptive for other functions, but compromised their abilities to compete aggressively. Specific traits that may provide advantages to small species in aggressive interactions included well-developed leg musculature and talons, enhanced flight acceleration and maneuverability, novel fighting behaviors, and traits associated with aggression, such as testosterone and muscle development. Traits that may have hindered larger species in aggressive interactions included the evolution of morphologies for tree trunk foraging that compromised performance in aggressive contests away from trunks, and the evolution of migration. Overall, our results suggest that fundamental trade-offs, such as those associated with body size, are more likely to break down over evolutionary time, changing the rules that govern species interactions and structure ecological communities.

## Introduction

Phylogenetic perspectives have changed the way we view ecological communities by incorporating evolutionary history into explanations of patterns of coexistence and resource use [1], [2]. Because closely-related species are more likely to share traits and be ecologically similar due to recent, shared ancestry, phylogenetic relationships may influence the degree to which species can coexist [1], [2]. One particular way that evolutionary relatedness can influence community structure is by altering the trade-offs that constrain species interactions and distributions [3], [4]. For example, small-sized animals benefit from reduced energetic and water requirements for survival and reproduction, reduced developmental times, reduced costs of locomotion, greater ability to quickly shed or absorb heat, greater maneuverability, and faster response time [5], [6]. However, small-sized animals typically lose aggressive contests for resources because larger animals generate greater force for a given acceleration, require a greater opposing force to overcome their inertia or change their momentum, and have greater muscle mass and strength, stronger defensive coverings, and larger and stronger traits used as weapons (e.g., teeth, bills, claws) [5], [7], [8]. Yet, the dominance of larger body size is not universal. Smaller species can overcome the advantages of large size in aggressive contests with the evolution of novel traits or trait values (e.g., weapons, enhanced maneuverability, or social behavior) that offset the disadvantage of small size (e.g., [9]–[11]). Such novel traits should accumulate over evolutionary time [3], [12], leading to the hypothesis that the advantage of large size in aggressive interactions should decline with evolutionary distance among the species.

Here, we test this hypothesis using data from 23,362 interactions among 246 species pairs, representing three phylogenetically and ecologically distinct groups of birds that have been studied extensively with respect to aggressive contests for shared food resources. These three groups are: 1) New World vultures (Accipitriformes: Cathartidae) and Old World vultures (Accipitriformes: Accipitridae) interacting at carcasses, 2) hummingbirds (Apodiformes: Trochilidae) interacting at nectar sources, and 3) antbirds (Passeriformes: Thamnophilidae) and woodcreepers (Passeriformes: Dendrocolaptidae) competing for invertebrates and small vertebrates fleeing from army ant swarms (Hymenoptera: Formicidae) [13].

## Materials and Methods

### Aggressive Interactions

**Figure 1:**
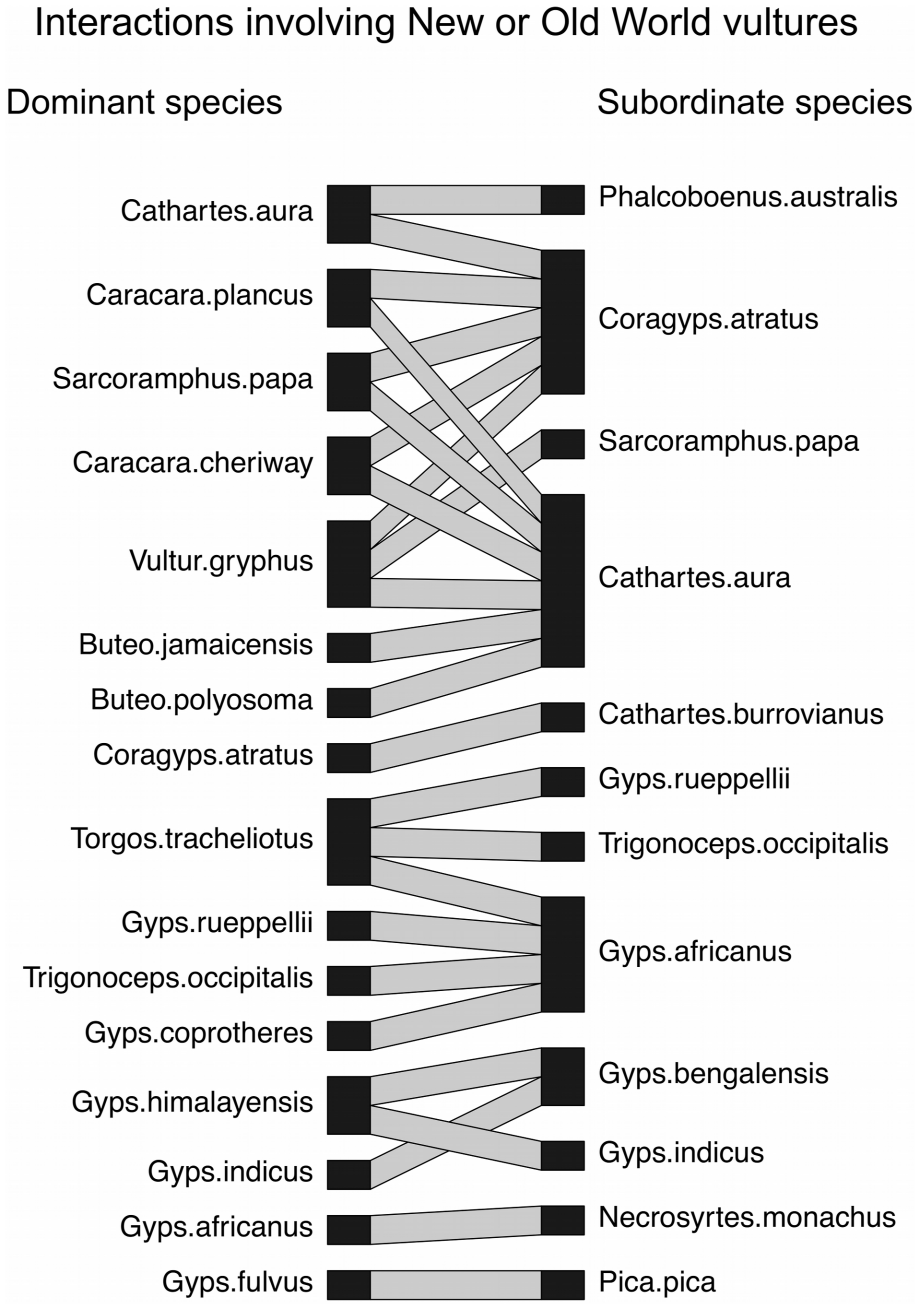
An interaction web for aggressive interactions involving New World vultures (Cathartidae) or Old World vultures (Accipitridae) at a carrion food source. Lines connect species pairs for which we include data on aggressive interactions in this study. Dominant species (left column) were defined as species winning the majority of aggressive interactions with the subordinate species (right column).

**Figure 2:**
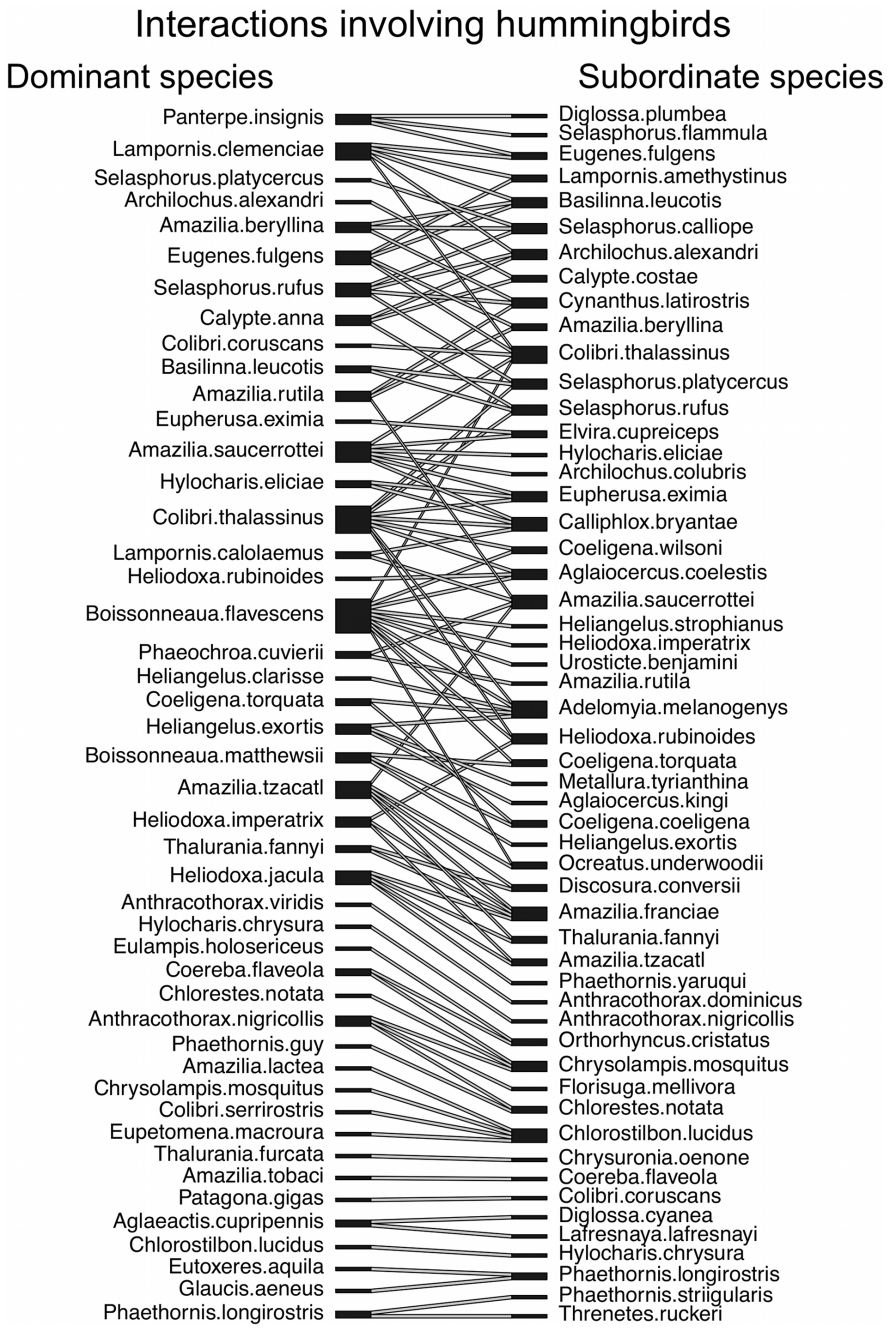
An interaction web for aggressive interactions involving hummingbirds (Trochilidae) at a nectar food source. Lines connect species pairs for which we include data on aggressive interactions in this study. Dominant species (left column) were defined as species winning the majority of aggressive interactions with the subordinate species (right column). One species pair (*Hylocharis chrysura* — *Thalurania furcata*) was omitted from this figure because each species won the same number of interactions with the other.

**Figure 3:**
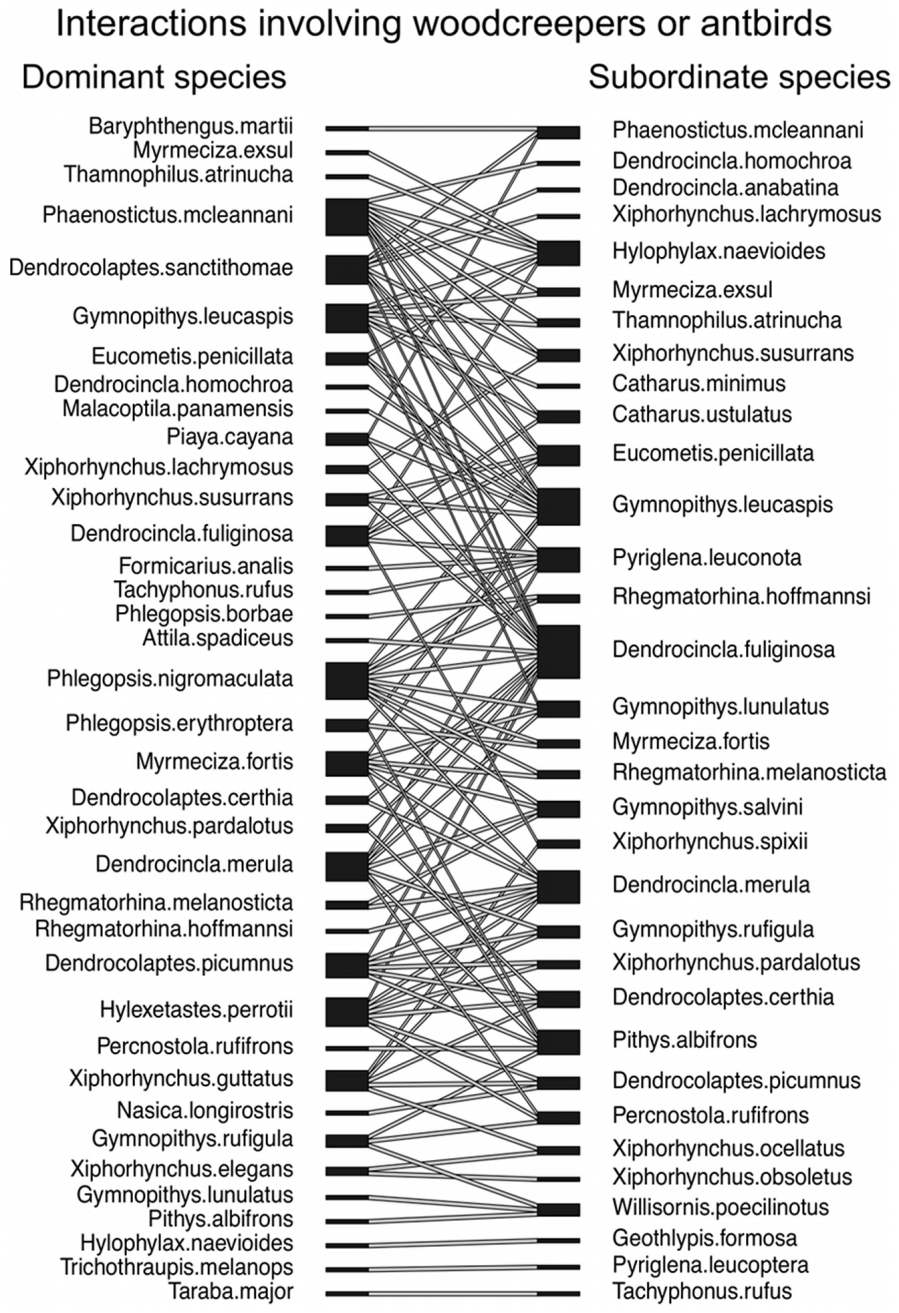
An interaction web for aggressive interactions involving antbirds (Thamnophilidae) or woodcreepers (Dendrocolaptidae) at army ant swarms, where they feed on prey flushed by the ants. Lines connect species pairs for which we include data on aggressive interactions in this study. Dominant species (left column) were defined as species winning the majority of aggressive interactions with the subordinate species (right column). One species pair (*Pyriglena leucoptera* — *Tachyphonus coronatus*) was omitted from this figure because each species won the same number of interactions with the other.

We compiled published quantitative data on aggressive interactions between our focal species (vultures, hummingbirds, and woodcreepers and antbirds) and any other species of bird, but restricted our analysis to pairs of species with at least 6 interactions where each interaction was won by one of the two species (Figures 1–3; Tables S1, S2). We compiled data on aggressive interactions that were associated with a shared resource (following [14]), including (a) chases, where one species actively pursued the other species, (b) supplants and displacements, where one species actively flew at, lunged, pecked, or otherwise aggressively engaged another species, causing the other species to retreat, and (c) physical attacks, where one species fought with another species (e.g., pecking, grabbing, hitting with wings, pinning to ground), resulting in the losing species retreating from the altercation [14]. We included only observations where one species was a clear winner over the other, as described in the original reference. We excluded observations that could be viewed as defense of eggs, young, or nests because these interactions did not involve a shared resource, and because the fitness costs of losing offspring are higher for the parent species. We excluded interactions that involved more than one individual of each of two species. When possible, we excluded interactions involving young birds; however, details of age were often unavailable. We supplemented our interaction dataset with our own unpublished natural history observations of hummingbirds interacting at feeders in South America.

Overall, we compiled data on 23,362 aggressive interactions among 246 species pairs (Tables S1, S2). In total, 145 different species were represented in our dataset, including 99 species as dominant (i.e., winning the majority of aggressive interactions with another species) and 99 species as subordinate (i.e., losing the majority of aggressive interactions with another species) (Figures 1–3). Twenty-seven pairs of interacting species were in the same genus, 157 pairs were in different genera but in the same family, and 62 pairs represented different taxonomic families. On average, each of the 145 species was represented in 3.4 pairwise interactions (range 1–18).

### Body Mass

Whenever possible, we obtained average adult mass for each species in our study from the same studies that provided the interaction data. Otherwise, we obtained adult mass data from areas as geographically close as possible to the populations where the interaction data originated. We calculated the mean mass of each species as the mean of males (mean male mass) and females (mean female mass), if males and females had different masses. We provide the sources for mass data for each species pair in Table S2.

### Genetic Distance

We compiled mitochondrial genetic sequence data from Genbank (accession numbers are provided in Text S1). Once aligned, we measured genetic distance between the sequences of focal species pairs using MEGA version 5.0 [15]. We calculated between-group mean Tamura-Nei genetic distances because this measure corrects for multiple substitutions at one site, incorporates differences in substitution rates between nucleotides, and does not assume equal nucleotide frequencies [16]. Species interactions lacking relevant genetic sequence data were excluded from our analysis.

We preferentially used the mitochondrial gene cytochrome *b* (cyt*b*) to estimate genetic distance between pairs of interacting species because this gene appears to evolve in a clock-like fashion in birds [17]. However, too few hummingbird species had cyt*b* sequences available, so we examined the mitochondrial gene NADH dehydrogenase subunit 2 (ND2) for hummingbirds. We aligned sequences of each gene with the same gene from the chicken (*Gallus gallus*) [18] using Clustal X [19], visually inspected the sequences using MacClade version 4.08 [20], and removed sequences that did not align with the chicken sequence. We included transitions and transversions, all codon positions, assumed uniform rates among sites and homogeneous patterns among lineages, and used pairwise deletion to address gaps or missing data [15]. We calculated genetic distances for each pair of interacting species when species were in the same taxonomic family. For species representing different families, we calculated the genetic distances incorporating all sequences of species in those families that were included in our study to improve the accuracy of our longer distance estimates.

### Statistical Tests

We conducted all of our statistical tests in R [21]. We tested the hypothesis that the importance of large size in determining the outcome of aggressive contests declined with genetic distance between interacting species using a linear mixed-effects model with sqrt{ln[(wins by dominant species+1)/(wins by subordinate species+1)]} as the response variable, {(mass of dominant species−mass of subordinate species)/[(mass of dominant species+mass of subordinate species)/2]} and genetic distance as predictors within a saturated model, and group (vultures, hummingbirds, or antbirds/woodcreepers) as a random factor, with a Gaussian distribution using the R package nlme [22]. The dominant species was defined as the species that won the majority of aggressive contests. We also tested if smaller bird species were more likely to be dominant when interacting with a more distantly-related species using a generalized linear mixed model with the response variable equal to 0 if the larger species was dominant, and 1 if the smaller species was dominant, genetic distance and number of interactions between species as predictors in a saturated model, and group (vultures, hummingbirds, or antbirds/woodcreepers) as a random factor, with a binomial distribution using the R package lme4 [23]. In our generalized linear model, we standardized both genetic distance and the number of interactions between species prior to analysis by subtracting the mean and dividing by 2 standard deviations, using the *rescale* command in the R package *arm* [24]. Two species comparisons were excluded from analyses because each species won the same number of aggressive encounters, and thus we could not designate a dominant and subordinate species.

We used a mixed models approach with group (vultures, hummingbirds, woodcreepers/antbirds) as a random factor because interactions between species within vultures, hummingbirds, and woodcreepers/antbirds often lacked independence (e.g., one species interacted with more than one other species; Figures 1–3). In contrast, we had no overlap of species across our three groups. We also ran our analyses including either the dominant species or the subordinate species as a random factor, nested within group, to ensure that our results were not influenced by one or a few species that were small and dominant or large and subordinate. We did not run our analysis with both dominant and subordinate species as random factors because each interaction among species pairs was unique.

We first ran the saturated model with different random slopes and intercepts and chose the best model as the model with the lowest Akaike Information Criterion (AIC) value [25]. We then checked the fit of the best saturated model following [25]. If saturated models did not adequately fit the data (e.g., residuals were significantly different from normal for linear mixed-effects models), we either modeled heterogeneity and assessed new model performance using AIC values and improved model fit [25], or transformed dependent or independent variables to improve model fit. We then ran models that incorporated all possible combinations of predictor variables using the R package MuMIn [26], and compared the fit among models using AIC values adjusted for small sample sizes (AICc). We identified the best-fit model as the model with the lowest AICc score. We ran our generalized linear mixed models using both the R packages lme4 [23] and MASS [27] to ensure that our results were consistent across packages [25].

We ran two additional generalized linear models to test our hypothesis, with the same predictor and response variables as in the saturated linear mixed-effects and generalized linear mixed models, but with group (vultures, hummingbirds, antbirds/woodcreepers) entered as a predictor variable in a saturated model (without random effects). This model allowed us to test for variation in the effects of genetic distance and body mass on the outcome of aggressive interactions between our 3 focal groups. The model, however, assumes that interactions among species are independent — an assumption that was violated by some species interacting with multiple species within groups. We ran models incorporating all possible combinations of predictor variables and checked the fit of our models as in our previous models.

### Number of Interactions among Species Pairs

The number of aggressive interactions over shared resources are expected to decline as species diverge over evolutionary time because more distantly-related species, on average, are ecologically more distinct [8], [28]. Small species could win more interactions with larger species when interactions are rare if they can bluff or if species are more inclined to back down when the other species is poorly known. We did not include the number of interactions among species pairs in our main linear mixed-effects model because the number of interactions was already incorporated into the dependent variable. We did, however, include the total number of interactions among species pairs in our generalized linear mixed model.

The total number of interactions in our generalized linear mixed model may be biased by the inclusion of different studies with different effort and sample sizes. Thus, we ran the analysis again including each study as a random factor nested within group (vultures, hummingbirds, woodcreepers/antbirds). By including study as a random factor, we could address if the number of interactions among species pairs within studies influenced our main results. We included only studies that had at least 2 species pairs, and included only species pairs with at least 6 interactions within a study. We again included the number of interactions and genetic distance as predictors in a saturated model, with the response variable equal to 0 if the larger species was dominant, and 1 if the smaller species was dominant, and a binomial distribution using the R package lme4 [23]. For all analyses, the number of interactions was transformed to improve model fit, as: n = ((log(log(number of interactions)))∧0.1).

## Results

**Figure 4:**
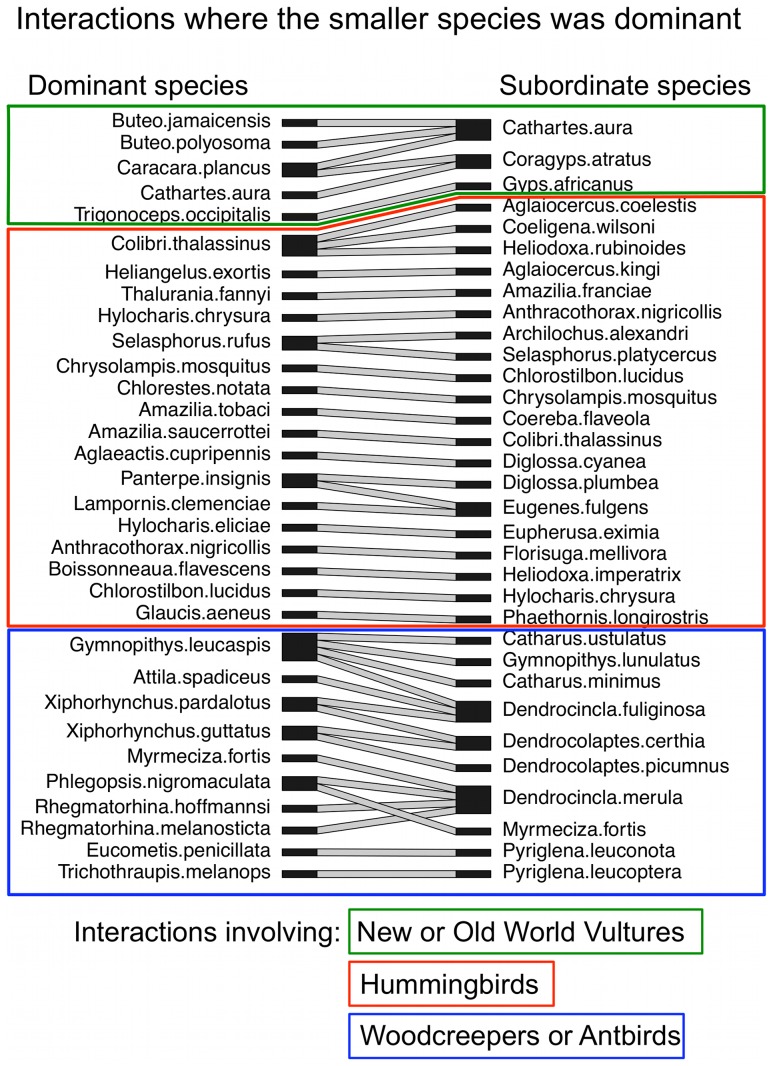
An interaction web for aggressive interactions where the smaller (lighter) species was dominant to the heavier species. Each interacting species pair where the smaller species was dominant is connected by a line (*n* = 43 species pairs). Dominant species (left column) were defined as species winning the majority of aggressive interactions with the subordinate species (right column). Species pairs that include a vulture are indicated by a green box (top), hummingbird by a red box (middle), and antbird or woodcreeper by a blue box (bottom).

The larger species won the majority of aggressive interactions with the smaller species (i.e., was behaviorally dominant) in 201 of 246 species pairs (81.7%). Smaller species were behaviorally dominant in 43 species pairs (17.5%) involving 32 different small, dominant species, and 33 different large, subordinate species (Figure 4). Wins and losses were equal in only two species pairs. In some cases where smaller species were behaviorally dominant, differences in weight were small and perhaps insignificant. In 32 species pairs, however, the larger species was>5% heavier than the smaller, dominant species, while 25 species pairs involved larger species that were>10% heavier than the smaller, dominant species.

**Figure 5:**
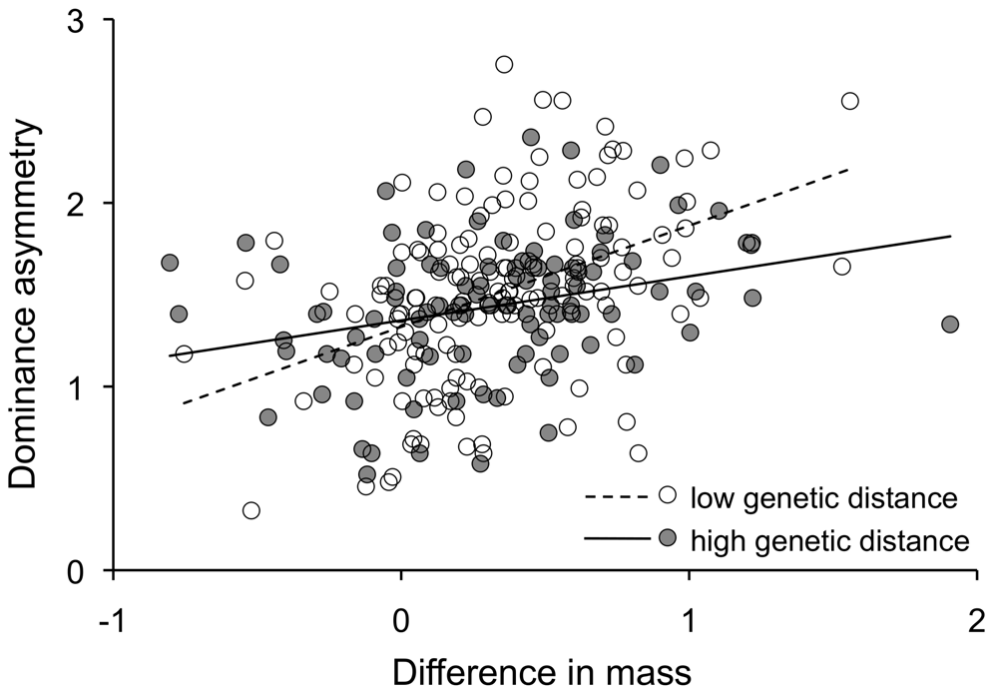
Relatively heavier bird species were more likely to win aggressive contests for resources (positive slope), but the advantage of large size in aggressive contests declined with genetic distance (shallower slope for high genetic distance). Dominance asymmetry (y-axis) = sqrt {ln ((wins by dominant species+1)/(wins by subordinate species+1))}. Difference in mass (x-axis) = (mass of dominant species−mass of subordinate species)/(average mass of dominant and subordinate species). Genetic distance groups are split by the midpoint value for the dataset (low = 0.006–0.179; high = 0.180–0.352).

**Figure 6:**
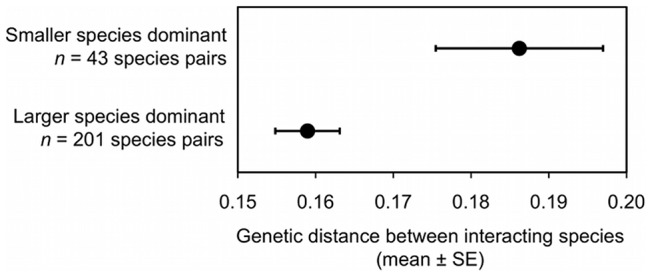
Smaller (lighter) bird species were more likely to be dominant (i.e., win the majority of aggressive contests for resources) when interacting with a more distantly-related species (greater genetic distance between species). Data are from aggressive interactions involving vultures at carcasses, hummingbirds at nectar sources, and woodcreepers and antbirds at army ant swarms.

The advantage of large size in aggressive contests was greatest among closely-related species and declined with increasing genetic distance (Figure 5; Tables 1,2; see Figure S1 for a 3-dimensional plot that includes difference in mass, genetic distance and dominance asymmetry). Similarly, smaller species were more likely to be dominant in aggressive interactions with distantly-related species (Figure 6; Tables 3,4). For example, among interacting species in the same genus, the smaller species was dominant in only 2 of 27 species pairs (7.4%), whereas among interacting species in different taxonomic families, the smaller species was dominant in 18 of 62 species pairs (29.0%). The decline in the importance of large size in aggressive interactions with increased genetic distance was evident in each of the three independent groups and did not differ significantly between them (Tables 5,6).

**Table 1:** Comparison of model fit for different linear mixed-effects models that include all possible combinations of predictor variables.

Model ranking	Intercept^1^	Difference in mass	Genetic distance	Interaction between difference in mass and genetic distance	df	logLik	AICc	delta AICc	weight
1	1.17	0.84	0.91	−2.46	6	−120.5	253.3	0.00	0.882
2	1.35	0.37	—	—	4	−124.9	258.0	4.72	0.083
3	1.31	0.37	0.24	—	5	−124.8	259.8	6.49	0.034
4	1.47	—2	—	—	3	−140.5	287.1	33.85	0.000
5	1.45	—	0.14	—	4	−140.5	289.1	35.83	0.000

Models tested the prediction that the advantage of large size in aggressive contests for resources declines with genetic distance among interacting bird species (*n* = 244 species pairs).

1 numbers for predictor variables are effect sizes.

2 long dash (—) indicates that the predictor variable was absent from the model.

**Table 2:** Results from the best-fit (lowest AICc) linear mixed-effects model testing the prediction that the advantage of large size in aggressive contests for resources declines with genetic distance among interacting bird species (*n* = 244 species pairs).

Fixed effects^1^	Estimate	SE	*t*	*df*	*P*
Intercept	1.17	0.10	11.91	238	<0.0001
Difference in mass^2^	0.84	0.17	4.91	238	<0.0001
Genetic distance	0.91	0.48	1.89	238	0.0598
Difference in mass^2^×Genetic distance	−2.46	0.84	−2.94	238	0.0036

1 dependent = sqrt{ln[(wins by dominant species+1)/(wins by subordinate species+1)]}; taxonomic group included as a random effect.

2 (mass of dominant species−mass of subordinate species)/(average mass of dominant and subordinate species).

**Table 3:** Comparison of model fit for different generalized linear mixed models (binomial distribution) that include all possible combinations of predictor variables.

Model ranking	Intercept^1^	Genetic Distance	Number of interactions	Interaction between genetic distance and number of interactions	df	logLik	AICc	delta AICc	weight
1	−1.60	0.89	—	—	3	−110.1	226.4	0.00	0.587
2	−1.61	0.91	0.07	—	4	−110.1	228.4	2.02	0.213
3	−1.58	0.96	0.00	0.76	5	−109.6	229.4	3.03	0.129
4	−1.54	—2	—	—	2	−113.6	231.3	4.88	0.051
5	−1.54	—	−0.10	—	3	−113.6	233.2	6.84	0.019

Models tested the prediction that smaller bird species were more likely to win aggressive contests for resources when interacting with a more distantly-related species (*n* = 244 species pairs).

1 numbers for predictor variables are effect sizes; genetic distance and number of interactions were standardized prior to analysis by subtracting the mean and dividing by 2 standard deviations.

2 long dash (—) indicates that the predictor variable was absent from the model.

**Table 4:** Results from the best-fit (lowest AICc) generalized linear mixed model (binomial distribution) testing the prediction that smaller bird species were more likely to win aggressive contests for resources when interacting with a more distantly-related species (*n* = 244 species pairs).

Fixed effects^1^	Estimate	SE	*z*	*P*
Intercept	−1.60	0.18	−9.08	<0.0001
Genetic distance^2^	0.89	0.35	2.59	0.0098

1 dependent = 0 if larger species was dominant, 1 if smaller species was dominant; taxonomic group included as a random effect.

2 standardized prior to analysis by subtracting the mean and dividing by 2 standard deviations.

**Table 5:** Comparison of model fit for different generalized linear models, with taxonomic group entered as a predictor that include all possible combinations of predictor variables.

Model ranking	Intercept^1^	Taxonomic group	Difference in mass	Genetic distance	Interaction between group and difference in mass	Interaction between group and genetic distance	Interaction between difference in mass and genetic distance	Interaction between group, genetic distance and mass	df	logLik	AICc	delta AICc	weight
1	1.08	+^2^	0.85	0.85	—	—	−2.53	—	7	−117.2	249.0	0.00	0.387
2	0.79	+	0.93	3.26	—	+	−2.92	—	9	−115.3	249.3	0.35	0.325
3	1.11	+	0.78	0.93	+	—	−2.88	—	9	−116.4	251.6	2.65	0.103
4	0.82	+	0.87	3.35	+	+	−3.32	—	11	−114.5	252.1	3.11	0.082
5	1.27	+	0.37	—	—	—	—	—	5	−121.9	254.0	5.07	0.031
6	1.17	—3	0.85	0.93	—	—	−2.44	—	5	−122.1	254.5	5.59	0.024
7	0.65	+	1.19	4.51	+	+	−5.35	+	13	−113.6	254.8	5.87	0.021
8	1.25	+	0.37	0.19	—	—	—	—	6	−121.8	255.9	6.99	0.012
9	1.28	+	0.32	—	+	—	—	—	7	−121.4	257.3	8.34	0.006
10	1.10	+	0.37	1.50	—	+	—	—	8	−120.8	258.3	9.31	0.004
11	1.35	—	0.39	—	—	—	—	—	3	−126.4	259.0	10.03	0.003
12	1.26	+	0.33	0.17	+	—	—	—	8	−121.3	259.3	10.32	0.002
13	1.31	—	0.39	0.23	—	—	—	—	4	−126.3	260.7	11.79	0.001
14	1.12	+	0.34	1.46	+	+	—	—	10	−120.4	261.7	12.76	0.001
15	1.41	+	—	—	—	—	—	—	4	−137.2	282.5	33.59	0.000
16	1.40	+	—	0.12	—	—	—	—	5	−137.2	284.6	35.62	0.000
17	1.29	+	—	1.09	—	+	—	—	7	−136.6	287.8	38.81	0.000
18	1.48	—	—	—	—	—	—	—	2	−143.5	291.1	42.19	0.000
19	1.47	—	—	0.09	—	—	—	—	3	−143.5	293.2	44.20	0.000

Models tested the prediction that the advantage of large size in aggressive contests for resources declines with genetic distance among interacting bird species (*n* = 244 species pairs).

1 numbers for predictor variables are effect sizes.

2 plus sign (+) indicates that the predictor variable (factor) was included in the model.

3 long dash (—) indicates that the predictor variable was absent from the model.

**Table 6:** Results from the best-fit (lowest AICc) generalized linear model, with taxonomic group included as a predictor variable, testing the prediction that the advantage of large size in aggressive contests for resources declines with genetic distance among interacting bird species (*n* = 244 species pairs).

Fixed effects^1^	Estimate	SE	*t*	*df*	*P*
Intercept^2^	1.08	0.11	9.70	243,238	<0.0001
Taxonomic group (hummingbirds)	0.05	0.09	0.58	243,238	0.56
Taxonomic group (antbirds/woodcreepers)	0.20	0.09	2.21	243,238	0.028
Difference in mass^3^	0.85	0.17	4.93	243,238	<0.0001
Genetic distance	0.85	0.49	1.76	243,238	0.08
Difference in mass^3^×Genetic distance	−2.53	0.84	−3.01	243,238	0.0029

1 dependent = sqrt{ln[(wins by dominant species+1)/(wins by subordinate species+1)]}.

2 intercept value for taxonomic group = vultures.

3 (mass of dominant species−mass of subordinate species)/(average mass of dominant and subordinate species).

The decline in the advantage of large size with increased genetic distance was not caused by variation in the number of interactions among species pairs (overall number of interactions: Tables 3,4; number of interactions within a study: Tables S3,S4). The number of interactions among species (overall, or within a study) also did not predict when small bird species would be dominant in aggressive interactions (overall number of interactions: Tables 3,4; number of interactions within a study: Tables S3,S4).

Including species (either the dominant species, or the subordinate species) as a random factor, nested within group, yielded similar results for both linear mixed-effects models (Table S5) and generalized linear mixed models (Table S6), with the interaction between genetic distance and body mass significant in all analyses. We also found similar results in linear mixed-effects models with interactions between *Gyps fulvus* and *Pica pica* (an outlier point) excluded from the dataset (interaction between genetic distance and body mass in our best-fit model, estimate = −2.21±0.86SE, *t* = −2.58 *df* = 237, *p* = 0.011; Figure S2).

## Discussion

Small animals flourish in many ecological settings, but they often suffer the recurrent cost of losing aggressive contests for resources with larger animals [5]–[8]. At the same time, numerous examples exist of smaller species dominating larger species (e.g., [9]–[11]), but the conditions leading to small species dominating larger species are poorly understood. Here we tested the hypothesis that the disadvantage of being small in aggressive interactions could be overcome over evolutionary time through the accumulation of novel traits that can counteract the advantages of being large. We found support for this hypothesis: larger species were dominant over smaller species during aggressive interactions for shared resources, but the advantage of a larger body size declined with increased evolutionary distance in vultures, hummingbirds, and antbirds/woodcreepers (Figure 5; Tables 1,2). Similarly, small species were more likely to win the majority of aggressive contests when they interacted with more distantly-related species (Figure 6; Tables 3,4). These results suggest that body size and phylogenetic distance jointly shape the outcome of aggressive interactions in birds, which in turn may influence the structure of bird communities.

While our results are consistent with the predictions of our hypothesis, our results are also consistent with three alternative hypotheses. Distantly-related species are, on average, less likely to share preferred resources [8], [28], which could explain the patterns observed here, if (i) large species were less willing to invest in aggressive encounters over less preferred resources with distantly-related smaller species, (ii) if the costs of losing an aggressive encounter are lower over less preferred resources, or (iii) if the context of aggressive interactions (e.g., age, experience, condition, hunger level, time of arrival or colonization) shift as resource preferences diverge, allowing some distantly-related species to win interactions that they would normally lose (e.g., [29]–[31]). If ecological similarity declines with genetic distance [8], [28], then these alternatives predict that behaviorally dominant species should lose a larger proportion of interactions with distantly-related subordinates, independent of differences in size. Our results did not support this prediction: while genetic distance was an important predictor of the outcome of aggressive interactions outside of its interaction with body size (Tables 1,2,5,6), dominant species won a greater proportion of their interactions with more distantly-related subordinate species, opposite to the predicted pattern. Detailed data on resource use also suggests that larger species did not lose aggressive contests over less preferred food (Text S2, Table S7), contrary to the predictions of (i) and (ii). While our data do not rule out the contribution of these alternative hypotheses to the decline in the importance of size in aggressive encounters among more distantly-related species, they suggest that these alternative hypotheses cannot explain the pattern by themselves.

### What traits offset the importance of size in aggressive interactions?

No study to date has quantified a general set of traits which predictably offset the advantages of large size in aggressive interactions. However, upon reviewing the focal studies used to generate the data in this study (Text S3), in addition to other studies, we find a diverse set of traits that play a key role in offsetting the importance of size in aggressive interactions among birds. These traits include the evolution of well-developed leg musculature and talons (raptors [32]), adaptations that enhance flight acceleration (hummingbirds [33], [34]) and maneuverability (hummingbirds [33]–[37], woodcreepers [38]–[40]), novel fighting behaviors (woodcreepers [38]–[40], grouse [41]), and traits directly associated with aggression, such as testosterone and muscle development (hummingbirds [29], [42], [43], woodcreepers [44], blackbirds [45], [46]), The evolution of social behavior, where smaller individuals perform coordinated attacks on individuals of larger species, may shift the outcomes of aggressive interactions among species (e.g., mammalian carnivores [8]), although we excluded interactions involving multiple individuals of each species in our study. In addition, intraspecific clustering, leading to high densities of subordinate species, can overwhelm individuals of dominant species, leading to dominants reducing their territory size or abandoning resources altogether (vultures [47], [48], hummingbirds [29], [49], blackbirds [45], [46]). In these cases, social coordination among individuals is unnecessary — simply a high density of subordinate relative to dominant individuals may increase the costs of aggressive defense of a resource for dominants. In all of these cases, the evolution of novel traits (or trait values) in smaller species were thought to allow them to overcome the costs of small size in aggressive contests with larger species.

Focal studies of species interactions also revealed cases where adaptations for other functions compromised the ability of large species to compete aggressively with smaller species — a mechanism that we did not predict. Traits that may have hindered larger species in aggressive interactions included the evolution of specialized morphologies for tree trunk foraging in woodcreepers [50] that in turn compromised their performance in aggressive contests away from trunks [44], [51], and the evolution of migratory behavior, that compromised performance in aggressive contests with resident species. In the case of woodcreepers, the specialized adaptations for tree climbing [50] enhanced their performance in aggressive contests with some woodcreeper species on tree trunks [38]–[40], but compromised their performance in aggressive interactions with antbirds, which typically occur on small saplings and branches [44], [50]–[52]. In the case of migration, a fundamental trade-off appears to constrain the ability of species to excel at both migration and performance in aggressive interactions simultaneously (Text S3), resulting in smaller resident species dominating larger migrant species across many different environments [14], [53]–[55]

Overall, observations of smaller species dominating larger species suggest that diverse adaptations in both the smaller and larger species may offset the disadvantage of small size in aggressive contests among species. While the evolution of novel traits in small species could lead to counter-adaptations in large species, and thus an evolutionary arms race, subordinate species (large or small) may reduce costs of aggressive interactions from dominant species in a myriad of ways, such as by using alternative resources (e.g., [47]), shifting resource use in space and time (e.g., [13], [56]), or even by mimicking dangerous species (e.g., mimicry of bumblebees by some subordinate hummingbirds; [33], [42]). Trade-offs and other evolutionary constraints are also likely to limit evolutionary arms races — most traits involved in interspecific aggression are used for other functions as well, and changes to these traits could influence organismal performance in many different ways. Most of our focal species also interacted with many species simultaneously (e.g., [43], [56]), creating diverse sources of selection that can influence patterns of co-evolution among species [57].

### Overcoming trade-offs over evolutionary time

Overall, our results suggest that the phylogenetic relationships among species can influence the rules that govern their interactions. Closely-related species typically share more traits in common [28], intensifying the importance of fundamental trade-offs that constrain their interactions and relative distributions. Thus, any advantages to being small must be balanced against the fitness cost of coexisting with larger, closely-related species [7], [58]. Over evolutionary time, however, novel traits may significantly alter the costs associated with trade-offs [3], creating new ecological opportunities and different patterns of community organization. Thus, a phylogenetic perspective of community ecology is important, not just for understanding causal processes or rules that structure communities [1], [2], but also for understanding when and how these rules can be broken over evolutionary time. These dynamic interactions provide an example of why phylogenetic perspectives are invaluable for our understanding of the structure and function of ecological communities [1], [2].

## Supporting Information

Figure S1 **A 3-dimensional plot illustrating the relationships between dominance asymmetry, difference in mass, and genetic distance among the focal species pairs in our study.** Dominance asymmetry = sqrt {ln ((wins by dominant species+1)/(wins by subordinate species+1))}. Difference in mass = (mass of dominant species−mass of subordinate species)/(average mass of dominant and subordinate species). Genetic distance is the Tamura-Nei genetic distance between interacting species for mtDNA.
(TIF)

Figure S2 **Relatively heavier bird species were more likely to win aggressive contests for resources (positive slope), but the advantage of large size in aggressive contests declined with genetic distance (shallower slope for high genetic distance).** Dominance asymmetry (y-axis) = sqrt {ln ((wins by dominant species+1)/(wins by subordinate species+1))}. Difference in mass (x-axis) = (mass of dominant species−mass of subordinate species)/(average mass of dominant and subordinate species). Genetic distance groups are split by the midpoint value for the dataset (low = 0.006–0.179; high = 0.180–0.352). Figure S2 is identical to Figure 5, except that an outlier point (interaction between *Gyps fulvus* and *Pica pica*) has been removed.
(TIF)

Table S1 **Description of variables for our dataset (Table S2).**
(TXT)

Table S2 **Dataset used in our study.**
(TXT)

Table S3 **Comparison of model fit for different generalized linear mixed models (binomial distribution) that include all possible combinations of predictor variables, controlling for variation in sample sizes of interactions across studies.** Models tested the prediction that smaller bird species were more likely to win aggressive contests for resources when interacting with a more distantly-related species.
(CSV)

Table S4 **Results from the best-fit (lowest AICc) generalized linear mixed model (binomial distribution), controlling for variation in sample sizes of interactions across studies.** The model tests the prediction that smaller bird species were more likely to win aggressive contests for resources when interacting with a more distantly-related species.
(CSV)

Table S5 **Results from the best-fit (lowest AICc) linear mixed-effects model, with dominant or subordinate species included as a random effect.** The model tested the prediction that the advantage of large size in aggressive contests for resources declines with genetic distance among interacting bird species (n = 244 species pairs).
(CSV)

Table S6 **Results from the best-fit (lowest AICc) generalized linear mixed model (binomial distribution), with dominant or subordinate species included as a random effect.** The model tested the prediction that smaller bird species were more likely to win aggressive contests for resources when interacting with a more distantly-related species (n = 244 species pairs).
(CSV)

Table S7 **Relative diet specialization among species pairs where the smaller species was dominant (i.e., won the majority of aggressive interactions).** Focal diets were the food source over which aggressive interactions occurred: carrion for interactions involving vultures, nectar for interactions involving hummingbirds, and prey flushed by army ant swarms for interactions involving woodcreepers and antbirds.
(CSV)

Text S1 **Genbank accession numbers for genetic sequences used in our study.**
(DOCX)

Text S2 **Patterns of resource use and preference in cases where small species dominated large species in aggressive contests.**
(DOCX)

Text S3 **A review of traits that have been proposed to explain why small species dominate large species of birds, focusing on specific cases in all three of our taxonomic groups.**
(DOCX)
