# Supplementary figures and images for: When David Beats Goliath: The Advantage of Large Size in Interspecific Aggressive Contests Declines over Evolutionary Time
Source: PLoS One. 2014 Sep 24;9(9):e108741. doi: 10.1371/journal.pone.0108741 (PMC4177554; doi:10.1371/journal.pone.0108741)

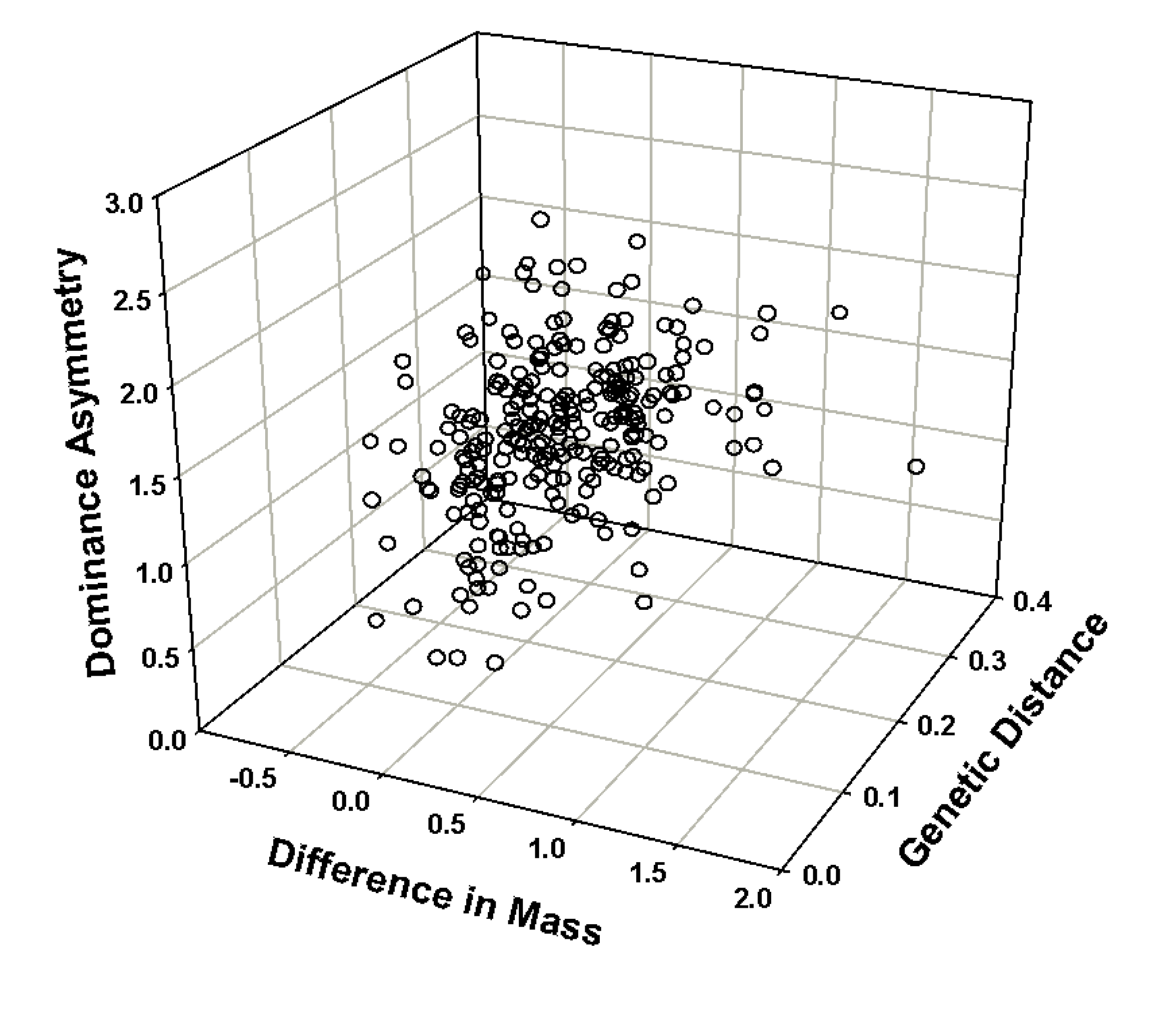

Supplement: Figure S1 — A 3-dimensional plot illustrating the relationships between dominance asymmetry, difference in mass, and genetic distance among the focal species pairs in our study. Dominance asymmetry = sqrt {ln ((wins by dominant species+1)/(wins by subordinate species+1))}. Difference in mass = (mass of dominant species−mass of subordinate species)/(average mass of dominant and subordinate species). Genetic distance is the Tamura-Nei genetic distance between interacting species for mtDNA. (TIF) [file pone.0108741.s001.tif]

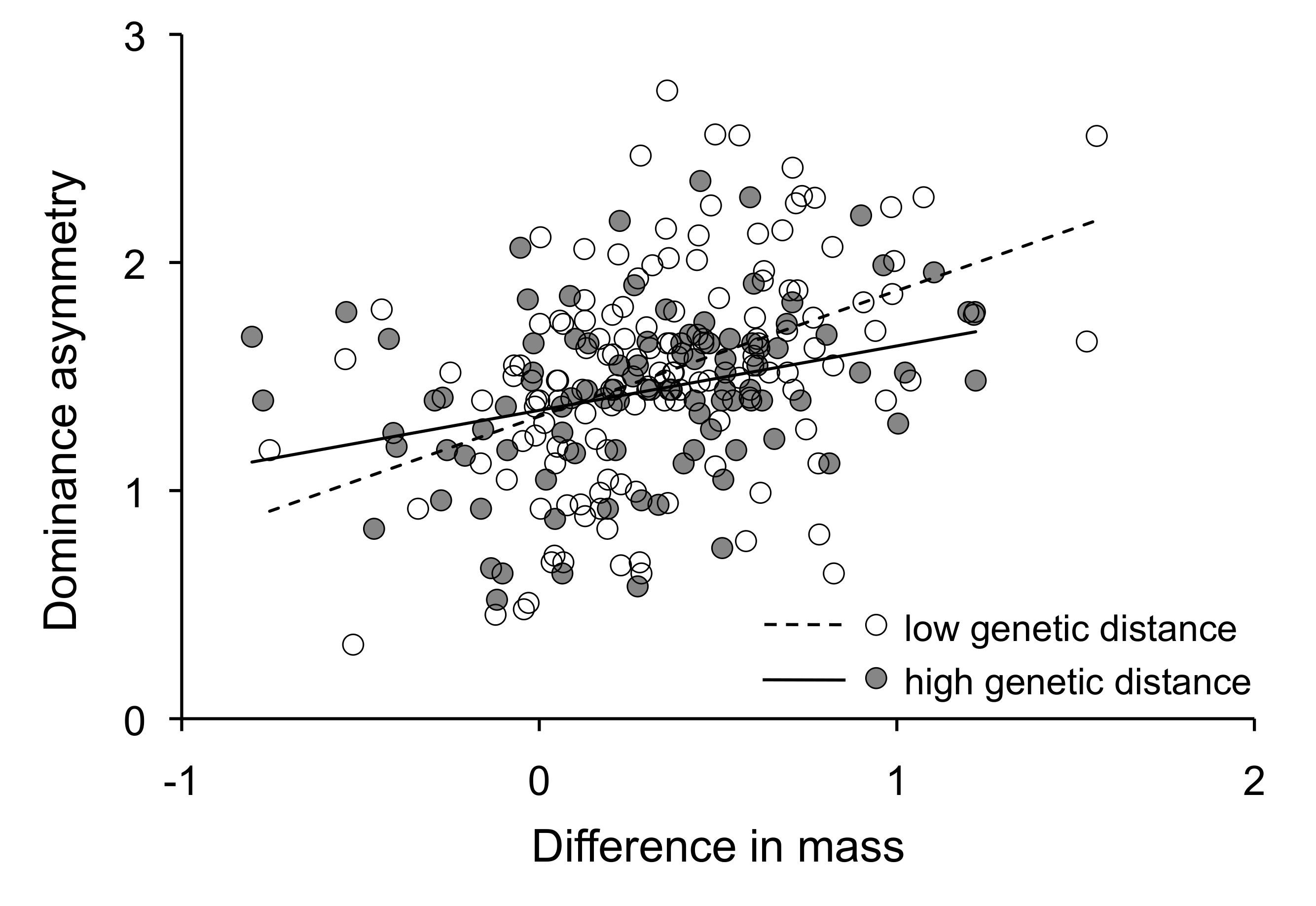

Supplement: Figure S2 — Relatively heavier bird species were more likely to win aggressive contests for resources (positive slope), but the advantage of large size in aggressive contests declined with genetic distance (shallower slope for high genetic distance). Dominance asymmetry (y-axis) = sqrt {ln ((wins by dominant species+1)/(wins by subordinate species+1))}. Difference in mass (x-axis) = (mass of dominant species−mass of subordinate species)/(average mass of dominant and subordinate species). Genetic distance groups are split by the midpoint value for the dataset (low = 0.006–0.179; high = 0.180–0.352). Figure S2 is identical to Figure 5, except that an outlier point (interaction between Gyps fulvus and Pica pica) has been removed. (TIF) [file pone.0108741.s002.tif]
