# Supplementary material for: When David Beats Goliath: The Advantage of Large Size in Interspecific Aggressive Contests Declines over Evolutionary Time
Source: PLoS One. 2014 Sep 24;9(9):e108741. doi: 10.1371/journal.pone.0108741 (PMC4177554; doi:10.1371/journal.pone.0108741)
Supplement: Text S1 — Genbank accession numbers for genetic sequences used in our study. (DOCX) [file pone.0108741.s010.docx]

**Text S1.** Genbank accession numbers for genetic sequences used in our study.

New World and Old World Vultures (cytochrome *b*): AF494341, AF494342, AY701182, AY701185, AY987258, AY987259, AY987260, AY987261, AY987263, AY987267, EU166984, EU233142, EU233143, EU233146, EU233147, EU496413, EU496425, EU496435, EU496441, EU496443, EU496447, EU496449, EU496453, EU496459, EU496466, GQ264784, GQ264785, GQ264798, GQ264799, U08944, U08946, X86743, X86760, X86761, X86762, X86763

Hummingbirds (NADH dehydrogenase subunit 2): AY115470, AY115472, AY830457, AY830459, AY830460, AY830461, AY830465, AY830466, AY830472, AY830473, AY830475, AY830476, AY830478, AY830481, AY830483, AY830486, AY830489, AY830491, AY830493, AY830496, AY830502, AY830504, AY830508, AY830509, AY830510, AY830511, AY830519, AY830522, AY830529, EU042521, EU042522, EU042523, EU042526, EU042529, EU042530, EU042531, EU042532, EU042533, EU042539, EU042541, EU042544, EU042552, EU042556, EU042557, EU042558, EU042562, EU042565, EU042580, EU042584, EU042589, EU042590, EU042593, EU418745, EU418757, EU418759, EU418761, EU543350, EU543352, EU543354, EU647937, EU647942, EU983395, EU983396, EU983416, EU983417, EU983418, EU983419, EU983428, EU983430, EU983434, EU983436, FJ175725, FJ175727, FJ175773, FJ175775, FJ903501, FJ903505, FJ903528, FJ903532, FJ903536, GU167203, GU167207, GU167214, GU167222, GU167231, GU167236, GU167244, GU167257, JF894062, JF905439, JN036616, JN036618, JN568603, JN568605, JQ445679, JQ445683, JQ445837, JQ445839

Woodcreepers and Antbirds (cytochrome *b*): AF383017, AF441631, AY065724, AY089791, AY089792, AY089793, AY089797, AY089800, AY089804, AY089807, AY089809, AY089817, AY089831, AY216855, AY370551, AY442991, AY442995, AY443002, AY504922, AY504924, AY676972, AY676976, AY676978, AY676979, AY676980, DQ157340, DQ157342, EF190605, EF190606, EF190607, EF202817, EF212895, EF212896, EF639949, EF639952, EF639963, EF639964, EF639977, EF639978, EF639980, EF639982, EF639983, EF639986, EF639995, EF639996, EF639997, EF639999, EF640001, EU166981, EU619788, EU619790, EU619802, EU619804, FJ799875, FJ799885, FJ799887, FJ799897, FJ799899, FJ799900, FJ804574, FJ804576, FJ804579, FJ804581, FJ899175, FJ899177, FJ899190, FJ899292, FJ899294, FJ899323, FJ899358, FJ899360, GU215176, GU215183, GU215187, GU215202, GU215234, GU215237, GU215244, GU215279, GU215280, GU215311, GU215350, HM165008, HM165010, JF276383, JN622092, JN622101, JN622102, JN622104.
