# Supplementary material for: When David Beats Goliath: The Advantage of Large Size in Interspecific Aggressive Contests Declines over Evolutionary Time
Source: PLoS One. 2014 Sep 24;9(9):e108741. doi: 10.1371/journal.pone.0108741 (PMC4177554; doi:10.1371/journal.pone.0108741)
Supplement: Text S2 — Patterns of resource use and preference in cases where small species dominated large species in aggressive contests. (DOCX) [file pone.0108741.s011.docx]

**Text S2.** Patterns of resource use and preference in cases where small species dominated large species in aggressive contests.

If larger species lost encounters with smaller species over less preferred food, then we might predict that most cases where the larger species was subordinate would involve small species that specialized on the focal food resource (carrion, nectar, or prey flushed by army ants) and larger species that used this resource only occasionally. Of the 43 species interactions where the smaller species was dominant, only 10 interactions involved specialists interacting with non-specialists (Table S7). Smaller species that specialized on focal resources dominated larger non-specialists in 3 species interactions, while smaller, non-specialist species dominated larger specialists in 7 species interactions (Table S7). These data do not support the hypothesis that that larger species were more likely to lose interactions over less preferred resources in our study.

Specific data from studies of interactions included in our analysis also do not support the alternative that larger species regularly lose at less preferred resources. For example, Prior and Weatherhead [S1] experimentally provided Turkey Vultures (*Cathartes aura*) carrion designed to match resource preferences of that species. Despite the fact that Turkey Vultures are carrion specialists and were feeding on their preferred food, vultures were displaced from carrion on 11 occasions by the smaller Red-tailed Hawk (*Buteo jamaicensis*) — a species that feeds on carrion only opportunistically [S1,S2]. When hawks fed at the carrion, vultures would sit nearby until the hawks left, and then resume feeding [S1], suggesting that the smaller Red-tailed Hawks were preventing Turkey Vultures from using a valued food resource.

Similarly, dominant territorial hummingbirds (e.g., *Amazilia saucerottei*, *Selasphorus rufus*) modified subordinate species' patterns of resource use through aggression, including larger subordinate species, even forcing them to leave the area entirely [S3,S4]. In the absence of dominant territorial species, subordinates shifted their resource use, increasing their use of resources to which they had been previously excluded [S3]. Even subordinate species with distinct ecological strategies (such as "trap-lining" species that visit many dispersed, small flower patches) shifted to high density flower patches when territorial species were temporarily absent [S3]. In the case of army ant swarms, *Catharus ustulatus* thrushes are displaced from swarms by the smaller antbird, *Gymnopithys leucapsis*, but converge on the swarms as soon as *Gymnopithys* leaves for other activities, such as preening ([S5]: page 203). Overall, the shifts in resource use in response to a behaviorally dominant species suggest that subordinates — large or small — are excluded from a high valued resource, and thus large species are not losing interactions over less preferred resources.

**Supplemental References**

**S1.** Prior KA, Weatherhead PJ (1991) Competition at the carcass: opportunities for social foraging by Turkey Vultures in southern Ontario. Canadian Journal of Zoology 69: 1550-1556.

**S2.** del Hoyo J, Elliott A, Sargatal J, eds. (1994) Handbook of the birds of the world. Volume 2. Lynx Edicions, Barcelona, Spain.

**S3.** Feinsinger P (1976) Organization of a tropical guild of nectarivorous birds. Ecological Monographs 46: 257-291.

**S4.** Kodric-Brown A, Brown JH (1978) Influence of economics, interspecific competition, and sexual dimorphism on territoriality of migrant Rufous Hummingbirds. Ecology 59: 285-296.

**S5.** Willis EO (1966) The role of migrant birds at swarms of army ants. Living Bird 5: 187-231.
