# Supplementary material for: When David Beats Goliath: The Advantage of Large Size in Interspecific Aggressive Contests Declines over Evolutionary Time
Source: PLoS One. 2014 Sep 24;9(9):e108741. doi: 10.1371/journal.pone.0108741 (PMC4177554; doi:10.1371/journal.pone.0108741)
Supplement: Text S3 — A review of traits that have been proposed to explain why small species dominate large species of birds, focusing on specific cases in all three of our taxonomic groups. (DOCX) [file pone.0108741.s012.docx]

**Text S3.** A review of traits that have been proposed to explain why small species dominate large species of birds, focusing on specific cases in all three of our taxonomic groups.

Studies of interactions among our focal species have identified traits that may offset the importance of size in aggressive encounters in each of our three focal groups. In interactions involving vultures, predatory raptors such as hawks and caracaras have evolved talons and related musculature to depredate their prey, and also use these traits during fights and aggressive contests within and among species [S1]. New World vultures, however, rarely act as predators, and have poorly developed talons and associated musculature [S1], putting them at a disadvantage in aggressive contests with more distantly related raptors, regardless of size.

In hummingbirds, aggressive, territorial species often exhibit traits (e.g., wing morphologies) that enhance maneuverability and aerodynamic power, both of which may provide advantages in competitive interactions [S2,S3]. In contrast, subordinate hummingbird species that typically forage on widely-spaced, small patches of flowers ("trapliners") typically exhibit traits that confer improved flight efficiency, but come at a cost of reduced acceleration, burst aerodynamic power and maneuverability [S2,S3]. While the exact traits involved, and the relative importance of maneuverability versus burst aerodynamic power, are not yet known [S3], adaptations that enhance flight performance in competitive interactions are thought to allow smaller species to dominate some larger species in aggressive contests for nectar (e.g., *Amazilia saucerottei* dominating *Colibri thalassinus*; ([S4]:page 492); *Selasphorus rufus* dominating *S. platycercus*; [S5]). More generally, the evolution of hovering flight, greater maneuverability, faster response time, and other traits associated with foraging and resource defense in hummingbirds [S6] appear to offset the disadvantage of small size in aggressive contests with many larger passerine birds (e.g., *Diglossa*) lacking these specialized locomotor abilities. The importance of maneuverability versus maximum short-term (burst) power among species may also change across environments, potentially explaining a reversal in competitive ability between interacting *Selasphorus* hummingbirds at low and high altitudes [S3].

In woodcreepers, well-developed morphological adaptations for tree trunk foraging, coupled with a novel fighting style, appear to provide a key advantage that allows *Xiphorhynchus woodcreepers*, such as *X. guttatus*, to dominate larger *Dendrocolaptes* woodcreepers (*D. picumnus* and *D. certhia*). While *Dendrocolaptes* woodcreepers are also adapted to tree trunk foraging, they commonly fly out (sally) after prey and are less proficient at moving quickly on trunks [S7]. The faster *Xiphorhynchus* woodcreepers were able to hitch rapidly up tree trunks behind *Dendrocolaptes*, attack them from below, and then quickly retreat, using these behaviors to supplant and displace *Dendrocolaptes* at army ant swarms [S7-S9]. The prairie grouse *Tympanuchus phasianellus* similarly uses a novel fighting style to dominate the larger *T. cupido* and the even larger *Phasianus colchicus* in aggressive contests [S10]. While *T. cupido* and *P. colchicus* jump in the air and throw up their legs and feet during battles, *T. phasianellus* commonly lower their heads, crouch their bodies, and dart forward, grabbing and holding the tail or rump feathers of their opponents from below, even pulling out the feathers on some occasions [S10]. This novel fighting technique appears to frighten and intimidate the larger species, who lost most aggressive contests with the smaller *T. phasianellus*, and then avoided further encounters [S10].

Across all three of our focal groups, selection for aggressive behavior (within or among species) may favor investment in testosterone, muscle mass, and other traits used in aggressive interactions, and these investments may offset the disadvantage of small size in some cases. For example, nectivorous birds that defend flowers ("territorial" species), or that rely on displacing territorial birds ("marauder" species), are notoriously aggressive within and among species — a behavior important for defense or usurpation of productive nectar resources ([S11]:page 95, [S12,S13]). While marauder species are often large-bodied (e.g., *Anthracothorax* spp.), territorial species (e.g., *Amazilia* spp.) are usually mid-sized for hummingbirds (4-5 g), but are nonetheless highly aggressive [S12] and are sometimes dominant to larger species (Figure 4). Higher levels of aggressiveness have also been invoked to explain the behavioral dominance of smaller species in woodcreepers (*X. susurrans* — *Dendrocincla fuliginosa* [S14] and blackbirds (Icteridae, *Agelaius phoeniceus* — *A. tricolor* [S15,S16]).

Two additional adaptations to overcoming the disadvantages of small size in aggressive interactions are social behavior and intraspecific clustering. Smaller individuals that perform coordinated attacks on individuals of larger species can often prevail, and this social behavior is thought to be important for shifting the outcomes of aggressive interactions among many groups (e.g., [S17,S18]). In addition to social behavior, several studies suggest that high densities of subordinate species can overwhelm individuals of dominant species, leading to dominants reducing their territory size or abandoning resources altogether (see for example, hummingbirds; [S11]:pages 59-60, [S19]; Old World vultures [S17]; New World vultures [S20]; blackbirds [S15,S16]). In these cases, social coordination among individuals is unnecessary — simply a high density of subordinate relative to dominant individuals may increase the costs of aggressive defense of a resource for dominants. In our study, we restricted our analyses to interactions among individuals, and thus coordinated social behavior was unlikely to influence our results. However, small species occurring at high densities may have caused large species to lose individual aggressive interactions in our study, either by reducing the benefits to the larger species of winning each interaction (thus encouraging them to retreat more quickly), or by exhausting larger individuals facing an abundance of heterospecific challengers (cf. [S11]:pages 59-60, [S19]).

While novel adaptations may allow species to overcome the disadvantage of small size, novel adaptations for other functions may also compromise a species' ability to win aggressive contests. For example, woodcreepers (Dendorcolaptidae) share specialized morphological adaptations for tree climbing (e.g., ossification of leg tendons, development of flexor muscles at the expense of extensor muscles, major changes to foot and tail morphologies [S21]) that appear to compromise their abilities in aggressive interactions with antbirds, which typically occur away from large trunks [S14,S22]. In particular, the specialized morphology of woodcreepers restricts their ability to effectively move or grasp small saplings and other non-trunk perches [S14,S21,S22], allowing smaller, agile antbirds such as *G. leucapsis* to supplant and displace them [S14,S23]. In this case, the evolution of traits associated with tree trunk foraging in woodcreepers appears to have compromised their abilities in aggressive encounters with antbirds away from tree trunks.

Similar trade-offs have been invoked for the evolution of migration, where migrants are at a disadvantage in aggressive contests for resources with permanent residents (e.g., [S24-S26]), leading to many cases where smaller resident species dominate larger migrants ([S24,S26]; *Gymnopithys* — *Catharus* interactions in our study). The reasons why migrant species might be at a disadvantage to resident species in aggressive contests are not known, but hypotheses center on trade-offs between migration and aggressive ability. These hypotheses include: (1) migrant species have evolved to use variable and low quality resources (e.g., [S13]) that do not allow them to invest heavily in adaptations for territoriality and aggression, (2) energy or other resources used for migration (e.g., fat reserves) come at the expense of those available for aggressive interactions, (3) adaptations for migration, such as long wings, faster molt (leading to lower quality feathers), or small hind limbs [S27,S28] reduce performance in aggressive contests, (4) migrants must adapt to geographically variable selective pressures, putting them at a disadvantage with resident species that are adapted to local conditions, or (5) migrants lack experience and knowledge of local resources and risks, putting them at a disadvantage in aggressive interactions with permanent residents. All of these hypotheses suggest plausible mechanisms whereby migration reduces the ability of species to win aggressive contests for resources with resident species, regardless of body size.

**Supplemental References**

**S1.** del Hoyo J, Elliott A, Sargatal J, eds. (1994) Handbook of the birds of the world. Volume 2. Lynx Edicions, Barcelona, Spain.

**S2.** Feinsinger P, Chaplin SB (1975) On the relationship between wing disc loading and foraging strategy in hummingbirds. American Naturalist 109: 217-224.

**S3.** Altshuler DL (2006) Flight performance and competitive displacement of hummingbirds across elevational gradients. American Naturalist 167: 216-229.

**S4.** Feinsinger P, Colwell RK, Terborgh J, Chaplin SB (1979) Elevation and the morphology, flight energetics, and foraging ecology of tropical hummingbirds. American Naturalist 113: 481-497.

**S5.** Kodric-Brown A, Brown JH (1978) Influence of economics, interspecific competition, and sexual dimorphism on territoriality of migrant Rufous Hummingbirds. Ecology 59: 285-296.

**S6.** del Hoyo J, Elliott A, Sargatal J, eds. (1999) Handbook of the birds of the world. Volume 5. Lynx Edicions, Barcelona, Spain.

**S7.** Willis EO (1982) The behavior of Black-banded Woodcreepers (*Dendocolaptes picumnus*). Condor 84: 272-285.

**S8.** Willis EO (1983) Cis-Andean *Xiphorhynchus* and relatives (Aves, Dendrocolaptidae) as army ant followers. Revista Brasileira de Biologia 43: 133-142.

**S9.** Willis EO (1992) Comportamento e ecologia do arapaçu-barrado *Dendrocolaptes certhia* (Aves, Dendrocolaptidae). Boletim do Museu Paraense Emílio Goeldi, Série Zoologia 8: 151-216.

**S10.** Sharp WM (1957) Social and range dominance in gallinaceous birds — pheasants and prairie grouse. Journal of Wildlife Management 21: 242-244.

**S11.** Stiles FG (1973) Food supply and the annual cycle of the Anna Hummingbird. University of California Publications in Zoology 97: 1-109.

**S12.** Feinsinger P, Colwell RK (1978) Community organization among neotropical nectar-feeding birds. American Zoologist 18: 779-795.

**S13.** Feinsinger P, Swarm LA, Wolfe JA (1985) Nectar-feeding birds on Trinidad and Tobago: comparison of diverse and depauperate guilds. Ecological Monographs 55: 1-28.

**S14.** Willis EO (1972) The behavior of Plain-brown Woodcreepers, *Dendrocincla fuliginosa*. Wilson Bulletin 84: 377-420.

**S15.** Orians GH (1961) The ecology of blackbird (*Agelaius*) social systems. Ecological Monographs 31: 285-312.

**S16.** Orians GH, Collier G (1963) Competition and blackbird social systems. Evolution 17: 449-459.

**S17.** König C (1983) Interspecific and intraspecific competition for food among Old World vultures. In Vulture biology and management (Wilbur SR, Jackson JA, eds.), pp. 153-171. University of California Press, Berkeley, California, USA.

**S18.** Donadio E, Buskirk SW (2006) Diet, morphology, and interspecific killing in Carnivora. American Naturalist 167: 524-536.

**S19.** Lyon DL (1976) A montane hummingbird territorial system in Oaxaca, Mexico. Wilson Bulletin 88: 280-299

**S20.** Wallace MP, Temple SA (1987) Competitive interactions within and between species in a guild of avian scavengers. Auk 104: 290-295.

**S21.** del Hoyo J, Elliott A, Christie DA, eds. (2003) Handbook of the birds of the world. Volume 8. Lynx Edicions, Barcelona, Spain.

**S22.** Willis EO (1979) Behavior and ecology of two forms of White-chinned Woodcreepers (*Dendrocincla merula*, Dendrocolaptidae) in Amazonia. Papéis Avulsos de Zoologia 33: 27-66.

**S23.** Willis EO (1967) The behavior of Bicolored Antbirds. University of California Publications in Zoology 79: 1-132.

**S24.** Willis EO (1966) The role of migrant birds at swarms of army ants. Living Bird 5: 187-231.

**S25.** DesGranges J-L, Grant PR (1980) Migrant hummingbirds' accommodation into tropical communities. In Migrant birds in the Neotropics: ecology, behavior, distribution, and conservation (eds. Keast A, Morton ES), pp. 395-409. Smithsonian Institution, Washington, DC, USA.

**S26.** Leisler B (1992) Habitat selection and coexistence of migrants and Afrotropical residents. Ibis 134: S77-S82.

**S27.** Leisler B, Winkler H (2003) Morphological consequences of migration in passerines. In Avian migration (Berthold P, Gwinner E, Sonnenschein E, eds.), pp. 175-186, Springer-Verlag, Berlin, Germany.

**S28.** de la Hera I, Pérez-Tris J, Tellería JL (2009) Migratory behaviour affects the trade-off between feather growth rate and feather quality in a passerine bird. Biological Journal of the Linnean Society 97: 98-105.
